# Genotype III Saint Louis Encephalitis Virus Outbreak, Argentina, 2005

**DOI:** 10.3201/eid1211.060486

**Published:** 2006-11

**Authors:** Luis Adrián Diaz, Viviana Ré, Walter R. Almirón, Adrián Farías, Ana Vázquez, María Paz Sanchez-Seco, Javier Aguilar, Lorena Spinsanti, Brenda Konigheim, Andrés Visintin, Jorge García, Maria Alejandra Morales, Antonio Tenorio, Marta Contigiani

**Affiliations:** * Instituto de Virologia "Dr. J. M. Vanella," Cordoba, Argentina;; † Centro de Investigaciones Entomologicas de Cordoba, Cordoba, Argentina;; ‡ Instituto de Salud Carlos III, Majadahonda, Spain;; § Instituto Nacional de Enfermedades Virales Humanas "Dr. J. Maiztegui," Buenos Aires, Argentina

**Keywords:** Saint Louis viral encephalitis, Diseases outbreaks, Culex, Poultry, dispatch

## Abstract

Twenty-six years after it was last detected, Saint Louis encephalitis virus (SLEV) genotype III reemerged in 2005 in Córdoba, Argentina, where it caused an outbreak. Two genotype III SLEV strains were isolated from *Culex quinquefasciatus*. A 71.43% prevalence for neutralizing antibodies was found in domestic fowl in the homestead of a patient with encephalitis.

Saint Louis encephalitis virus (SLEV; genus Flavivirus, family Flaviviridae) emerged in Córdoba Province, Argentina, in 2002. A single case of human encephalitis occurred (*1*). An outbreak of SLEV with 47 laboratory-confirmed cases, 9 fatal, occurred in Córdoba Province in 2005 (*2*). SLEV is widely distributed in the United States and in Central and South America, maintained in transmission cycles involving Culex mosquitoes and various birds (*3*). According to serologic data, SLEV is distributed throughout Argentina, including subtropical provinces in the north to the cold temperate province of Rio Negro in the south. Sporadic symptomatic cases of Saint Louis encephalitis (SLE) have been reported since 1964 (*4*). SLEV strains have been isolated from Culex mosquitoes, rodents, and febrile humans. Serologic evidence of natural infection has been reported in horses, goats, cattle, and wild and domestic birds (*4*). To investigate the etiology of the human encephalitis outbreak, we sought to detect and characterize a viral agent from mosquitoes and evaluate prevalence of SLEV-neutralizing antibodies in domestic birds in Córdoba.

## The Study

During a human encephalitis outbreak in February 2005, we collected adult mosquitoes and blood-sampled domestic geese and chickens at an urban residence of a patient with confirmed SLE. In 1 night with light traps, we collected 393 mosquitoes: Aedes aegypti (2.8%), Cx. interfor (13%), Cx. quinquefasciatus (84%), and Ochlerotatus albifasciatus (0.2%). Seven pools of female mosquitoes were organized and processed as previously described (*5*). A Flavivirus-generic reverse transcription (RT)–PCR assay was used to detect flavivirus-infected mosquito pools, and cDNA amplicons were sequenced as previously described (*6*), resulting in 3 SLEV-positive pools of unengorged females: pools 4005 and 4006 of Cx. quinquefasciatus and 4002 of Cx. interfor (GenBank accession nos. DQ232620, DQ232621, and DQ232619, respectively). An aliquot of 0.1 mL of each positive pool was injected onto a Vero cell monolayer, and 2 SLEV strains, CbaAr-4005 and CbaAr-4006, were isolated from the 2 Cx. quinquefasciatus pools. Both strains required 4 blind passages after 6 days of incubation in Vero cells until cytopathic effect was observed on day 6 postinjection. The harvested supernatant and cells of the fourth passage contained 6 log_10_/mL PFUs. These 2 strains were reisolated from the original mosquito pools with the same technique.

To characterize the isolated SLEV strains, their E genes were sequenced after RT-nested-PCR amplification with primers SLE-841S 5´-GGTTTTGCCGCAATCCTGGNTAYGC-3´, SLE-869S 5´-AGTTGCGCTGGCGATTGGNTGGATG-3´, SLE-2546AS 5´-GAAATACTTGTAGTCACTCRTCCAC-3´, and SLE-2541AS 5´-ACTTGTAGTCACTCTTCCAYTTYTC-3´. The phylogenetic analysis was conducted with MEGA version 3.0 (*7*). Sequences were aligned with 71 other SLEV sequences available in GenBank and 3 other related flaviviruses as outgroups (WNV M12294, JEV M18370, and MVEV AF161266). Isolated viral strains were categorized in genotypes by using the classification proposed by Kramer and Chandler (*8*).

The 3 sequences derived from the positive mosquito pools (4002, 4005, and 4006) were identical except for 8 silent substitutions (among 87 nt analyzed) and were closely homologous to SLEV sequence AF013416, with a high bootstrapping value (999/1,000). Subsequently, the entire E glycoprotein gene was sequenced from the 2 cultured isolates (GenBank accession nos. DQ385451 and DQ385450), and a phylogenetic tree was derived (Figure). The closest related GenBank sequence was AF205490 (bootstrap value 999/1,000), corresponding to the 79V2533 strain of SLEV isolated from a pool of Culex mosquitoes collected in Santa Fe Province, Argentina, in 1979. Both strains we isolated, therefore, belong to the genotype III described by Kramer and Chandler (*8*).

**Figure:**
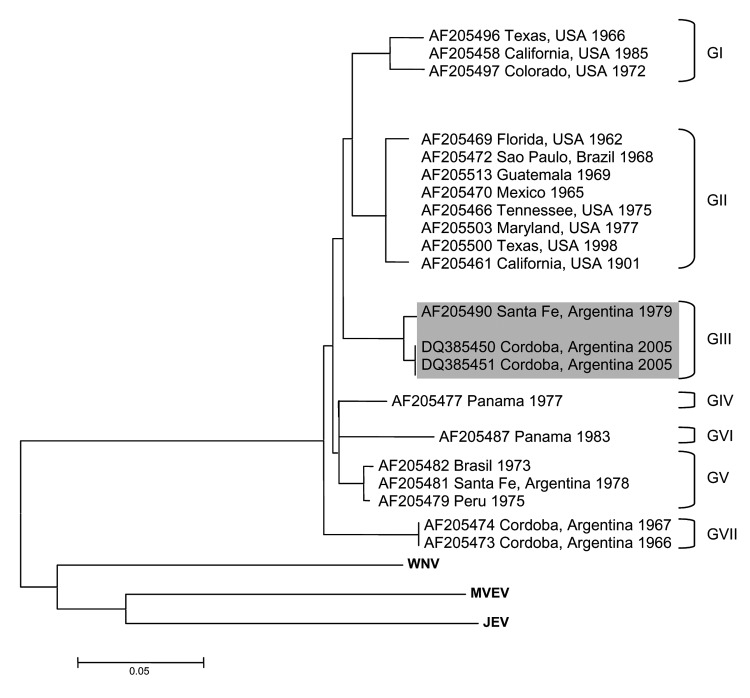
Consensus tree of the maximum parsimony analyses of Saint Louis encephalitis virus and other related flavivirus E glycoprotein genes. Shading indicates the genotype III to which the new viral strain belongs. West Nile virus (WNV), Japanese encephalitis virus (JEV) and Murray Valley encephalitis virus (MVEV) are used as outgroups. Scale bar indicates number of nucleotide differences.

Blood samples (0.2 mL) were taken from the jugular vein (chicks) or brachial vein (hens, geese) with a 27-gauge 3/8-inch needle attached to a 1-mL syringe and added to 0.9 mL sterile phosphate-buffered saline, for an ≈1:10 dilution of serum. Sera were tested for neutralizing antibodies by using the plaque-reduction neutralization test (*9*). Neutralizing antibodies against SLEV were detected in both geese and chickens (Table).

**Table:** Saint Louis encephalitis virus neutralizing antibody titers detected in domestic birds*

Host	Samples P/T	NtAb prevalence (%)	Age (mo)	Range in NtAb titer
*Anser anser* (goose)	4/5	80	>12	1,280†
*Gallus gallus* (chicken)	3/3	100	<3	1,280†
	8/13	62	>12	20–1,280

*At residence of a patient with Saint Louis encephalitis, during outbreak in Córdoba, Argentina, February 2005. P/T, number of positive samples/total samples analyzed; NtAb, neutralizing antibodies. †There was no NtAb range in these 2 cases. All positive sera had the same titer.

## Conclusions

The Córdoba outbreak in 2005 represents the first reported SLE outbreak in Central and South America. Before 2005, the only recorded outbreak of human encephalitis caused by flaviviruses in this region was in 1975 in Brazil, which was attributable to Rocío virus (*10*). The finding of genotype III SLEV strains in Córdoba Province indicates an extension of the distribution of this genotype to the central region of Argentina (*4*). The year of introduction of genotype III remains unknown; previously, genotype VII strains CorAn9124 and CorAn9275 circulated in Córdoba Province (*4*).

The reasons for the reemergence of SLEV genotype III in Argentina 26 years after it was last detected are unknown. Possible associated factors are mosquito species communities' species composition and relative abundance, climate, and avian host abundance and immunity. No investigation was conducted until recently to elucidate the SLEV transmission cycles in Argentina. Cx. quinquefasciatus is probably an SLEV vector, according to studies of vector competence, population abundance of mosquitoes, and viral isolations in Argentina (*5**,**11*). Our isolation of SLEV from Cx. quinquefasciatus during the 2005 outbreak in Córdoba, and its higher abundance compared with other mosquito species, suggests its role as a vector in the urban transmission cycle of SLEV. The role of Cx. interfor as a SLEV vector is unknown. This report represents the first detection of SLEV-infected Cx. interfor mosquitoes. At this time, no epidemiologic data for arboviral diseases associated with this mosquito species have been reported.

High susceptibility of avian hosts in the city of Córdoba for SLEV infection was confirmed in 2004 when <1% of free-ranging wild birds circulated neutralizing antibodies (L.A. Diaz, unpub. data). The high neutralizing antibody titers we detected in chickens <3 months of age indicated recent infection and support the hypothesis that SLEV was responsible for the simultaneous outbreak.

While the specific avian amplifying hosts involved in the Córdoba outbreak remain unknown, important amplifiers would include competent reservoir hosts that are abundant and frequently exposed to infectious mosquito bites (*12*). Based on abundance alone, some possible candidates for avian reservoirs in Córdoba would include chickens, eared doves (Zenaida auriculata), Picui ground doves (Columbina picui), house sparrows (Passer domesticus), rufous horneros (Furnarius ruffus), great kiskadee (Pitangus sulfuratus), and others. Eared doves are competent amplifying hosts (*13*). Our study indicated high exposure rates in chickens. While adult chickens are generally incompetent for SLEV strains, higher viremia levels develop in baby chicks, which would probably be competent hosts (*14*).

Finally, the reemergence of SLEV in Córdoba represents an opportunity to study the ecology of this virus. Further studies are needed on vector competence for local strains of Cx. quinquefasciatus and Cx. interfor and on the reservoir competence of the bird species mentioned above.

## References

[1] Spinsanti LI, Basquiera A, Bulacio S, Somale V, Kim SC, Re VE, St. Louis encephalitis in Argentina: the first case reported in the last seventeen years. Emerg Infect Dis. 2003;9:271–3. 12604006 10.3201/eid0902.020301 PMC2901953

[2] Spinsanti LI, Glatstein N, Arselán S, Diaz LA, Ré V, Aguilar J, Aspectos clínico-epidemiológicos de un brote por *Flavivirus* detectado en Córdoba, Argentina en el año 2005. Rev Argent Microbiol. 2005;7(S1):27.

[3] Reisen WK. Epidemiology of St. Louis encephalitis virus. Adv Virus Res. 2003;61:139–83. 10.1016/S0065-3527(03)61004-3 14714432

[4] Sabattini MS, Avilés G, Monath TP. Historical, epidemiological and ecological aspects of arbovirus in Argentina: *Flaviviridae, Bunyaviridae* and *Rhabdoviridae*. In: Travassos da Rosa APA, Vasconcelos PFC, Travassos da Rosa JFS, editors. An overview of arbovirology in Brazil and neighboring countries. Belem (Brazil): Instituto Evandro Chagas; 1998. p. 113–34.

[5] Diaz LA, Almiron WR, Ludueña Almeida F, Spinsanti LI, Contigiani MS. Vigilancia del virus Encefalitis de San Luis y mosquitos (Diptera: Culicidae) en la Provincia de Córdoba, Argentina. Entomol Vectores. 2003;10:551–66. Available from http://www.ugf.br/editora/revistas/entomologia/eyv2003/art14.pdf

[6] Sanchez-Seco MP, Rosario D, Domingo C, Hernandez L, Valdes K, Guzman MG, Generic RT-nested-PCR for detection of Flaviviruses using degenerated primers and internal control followed by sequencing for specific identification. J Virol Methods. 2005;126:101–9. 10.1016/j.jviromet.2005.01.025 15847925

[7] Kumar S, Tamura K, Nei M. MEGA3: integrated software for molecular evolutionary genetics analysis and sequence alignment. Brief Bioinform. 2004;5:150–63. 10.1093/bib/5.2.150 15260895

[8] Kramer LD, Chandler LJ. Phylogenetic analysis of the envelope gene of St. Louis encephalitis virus. Arch Virol. 2001;146:2341–55. 10.1007/s007050170007 11811684

[9] Early E, Peralta PH, Johnson KM. A plaque neutralization method for arboviruses. Proc Soc Exp Biol Med. 1967;25:741–7. 10.3181/00379727-125-32194 15938255

[10] de Souza Lopes O, de Abreu Sacchetta L, Coimbra TL, Pinto GH, Glasser CM. Emergence of a new arbovirus disease in Brazil. II. Epidemiologic studies on 1975 epidemic. Am J Epidemiol. 1978;108:394–401. 727209 10.1093/oxfordjournals.aje.a112637

[11] Mitchell CJ, Monath TP, Sabattini MS. Transmission of St. Louis encephalitis virus from Argentina by mosquitoes of the *Culex pipiens* (Diptera: Culicidae) complex. J Med Entomol. 1980;17:282–5. 7401124 10.1093/jmedent/17.3.282

[12] Scott TW. Vertebrate host ecology. In: Monta TP, editor. The arboviruses: epidemiology and ecology. Vol I. Boca Raton (FL): CRC Press; 1988. p: 257–80.

[13] Occelli M, Díaz LA, Spinsanti LI, Ludueña Almeida F, Almirón WR, Contigiani MS. Capacidad de *Zenaida auriculata* como hospedador de cepas patógenas del virus Encefalitis de San Luis (Flavivirus). Rev Argent Microbiol. 2005;37(S1):33.

[14] Trent DW, Monath TP, Bowen GS, Vordam AV, Cropp CB, Kemp GE. Variation among strains of St. Louis encephalitis virus: basis for a genetic, pathogenetic and epidemiological classification. Ann N Y Acad Sci. 1980;354:219–37. 10.1111/j.1749-6632.1980.tb27969.x 6261645

